# Transitioning from WiFi 6 to WiFi 7: A Metrological Assessment of Human-Centric EMF Exposure and Multi-Link Operation (MLO) Dynamics

**DOI:** 10.3390/s26082479

**Published:** 2026-04-17

**Authors:** Andreea Maria Buda, David Vatamanu, Sergiu Iulian Andreica, Calin Munteanu, Simona Miclaus

**Affiliations:** 1 Doctoral School, Technical University of Cluj-Napoca, 400114 Cluj-Napoca, Romania; 2 Department of Communications, IT & Cyber Defense, “Nicolae Balcescu” Land Forces Academy, 550170 Sibiu, Romania; 3 Department of Electrical Engineering and Measurements, Technical University of Cluj-Napoca, 400114 Cluj-Napoca, Romania

**Keywords:** WiFi 7, Multi-Link Operation (MLO), EMF exposure, 4096-QAM, CCDF analysis

## Abstract

This paper presents a comprehensive experimental assessment of electromagnetic field (EMF) exposure dynamics during the transition from IEEE 802.11ax (Wi-Fi 6) to IEEE 802.11be (Wi-Fi 7). Using a human-centric experimental setup, we evaluate the impact of Wi-Fi 7’s core innovations—4096-QAM modulation, 320 MHz bandwidth, and Multi-Link Operation—under iPerf3-controlled high-traffic conditions. A key contribution of this study is the analysis of multi-client influence, comparing EMF emission profiles when one versus two devices are active. Our results reveal a significant paradigm shift: while Wi-Fi 7 generates higher near-field peaks (up to 955.92 mV/m in MLO mode at 20 cm) to sustain high-order modulation, it exhibits an aggressive spatial decay, with E-field intensity collapsing by up to 76.6% at one meter. We demonstrate that the transition from a single-client to a dual-client configuration significantly alters the stochastic nature of the field, increasing the probability of transient high-power events, as characterized by our Complementary Cumulative Distribution Function (CCDF) framework. The findings confirm that Wi-Fi 7’s performance gains are decoupled from long-range exposure; the high-intensity field remains strictly localized, providing a natural safety buffer. This study provides new experimental vista into how next-generation WLAN systems trade near-field strength for far-field safety, maintaining compliance with international limits while supporting multi-device gigabit connectivity.

## 1. Introduction

The rapid proliferation of data-intensive applications—ranging from 8K video streaming to low-latency augmented and virtual reality (AR/VR)—has pushed the capabilities of Wireless Local Area Networks (WLAN) to their theoretical limits. While IEEE 802.11ax (WiFi 6) marked a significant milestone by integrating multi-user technologies to enhance spectral efficiency in dense environments, it is the emergence of IEEE 802.11be (WiFi 7) that promises a definitive leap toward Extremely High Throughput (EHT) [1,2,3]. Designed to ensure deterministic performance for real-time industrial communications and immersive cloud services, WiFi 7 introduces a level of waveform complexity that necessitates a reassessment of traditional electromagnetic field (EMF) characterization. Performance-oriented analyses, highlighting both current status and open challenges, confirm substantial gains in throughput and latency compared to previous standards [2,3,4,5,6,7].

The technical cornerstone of WiFi 7 lies in a fundamental shift in signal architecture, characterized by the adoption of 4096-QAM modulation and a doubled channel bandwidth of 320 MHz [1,3,8,9]. While these advancements optimize spectral occupancy, they inherently alter the transmission dynamics. Higher-order modulation schemes are known to induce significant envelope fluctuations, leading to elevated Peak-to-Average Power Ratio (PAPR) values [8,9]. Furthermore, the introduction of Multi-Link Operation (MLO) represents a paradigm shift in resource management, allowing devices to aggregate or switch traffic across the 2.4, 5, and 6 GHz bands simultaneously [4,5,6,7]. From an electromagnetic perspective, MLO does not merely increase throughput; it redefines the temporal emission patterns and the instantaneous superposition of radiated fields.

Such complexity renders conventional average-power metrics insufficient for a comprehensive exposure assessment. To bridge this gap, the Complementary Cumulative Distribution Function (CCDF) has emerged as a critical statistical tool for evaluating signal peaks in wideband systems [10]. Building upon our previous research [11,12,13,14], which demonstrated the efficacy of CCDF-based frameworks in characterizing radiated power dynamics, this study argues that a statistical approach is essential to capture the “real-world” peak electric field strengths that traditional measurement protocols often overlook.

Despite the fact that WiFi exposure levels consistently remain below the thresholds established by the International Commission on Non-Ionizing Radiation Protection (ICNIRP) [15] and IEEE C95.1-2019 [16], the evolution of the “electromagnetic footprint” remains a subject of intense scientific interest [17,18,19]. Compliance methodologies and numerical predictions, such as those detailed in ITU-T Recommendation K.91 [20], provide robust safety margins; however, they are largely based on averaged field quantities. Earlier investigations in diverse settings, including schools [21] and realistic indoor environments [22], have established baseline exposure levels for previous WiFi generations.

However, recent investigations into human-centric network trade-offs [23] and successive wireless generations [13,24] indicate that waveform structure and traffic load can significantly influence instantaneous and percentile-based exposure metrics. There is a notable lack of experimental evidence regarding how WiFi 7, with its aggregated MLO traffic, modifies the statistical distribution of radiated fields under reproducible, high-load conditions [25].

Furthermore, the “human-centric” dimension is frequently missing from existing exposure models. In operational scenarios, the human body acts as a significant electromagnetic perturbator, inducing absorption, scattering, and shadowing effects that radically redistribute field intensity [26,27]. To address these limitations, this work provides a rigorous experimental characterization of WiFi 6 and WiFi 7 emissions within a fully anechoic environment.

Crucially, our methodology incorporates the physical presence of human operators within the chamber, thereby accounting for the complex reflection and absorption variables inherent to realistic use cases [24,27]. By utilizing controlled traffic generation and CCDF analysis for signal decomposition, we provide a comparative analysis of instantaneous peaks, percentile-based indicators, and time-averaged metrics. This study aims to refine the metrological framework for next-generation WLAN systems, offering experimentally grounded insights into the evolving electromagnetic environment of WiFi 7.

## 2. Materials and Methods

### 2.1. Anechoic Chamber Configuration

**Figure 1:**
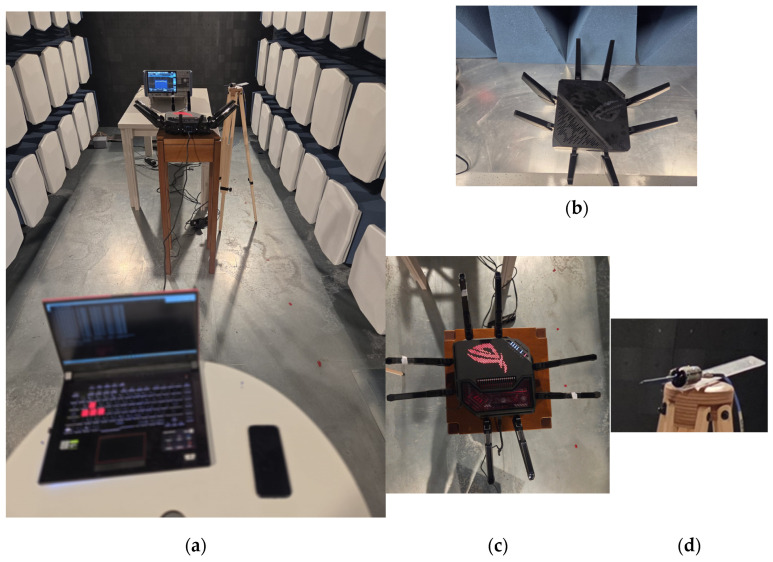
(**a**) System architecture: Spectrum analyzer, E-field probe on the tripod, router, laptop (server) and phone inside the anechoic chamber; (**b**) Wi-Fi 6 router; (**c**) Wi-Fi 7 router (**d**) detail of E-field probe.

To ensure the elimination of external interference and provide a highly repeatable multipath-free environment, all measurements were conducted inside a semi-anechoic chamber (Figure 1a). The facility has internal dimensions of [7.8 m × 3.8 m × 3.2 m]. The chamber is lined with high-performance pyramidal radio-frequency (RF) absorbers, providing a shielding effectiveness of better than 100 dB across the frequency range of interest (2.4 GHz to 6.2 GHz). All supporting structures within the chamber, including the test table and probe stands, were constructed from low-permittivity materials to minimize parasitic reflections.

This setup ensures that the measured electric (E)-field levels originate solely from the equipment under test (EUT), effectively isolating the experiment from ambient wireless noise.

### 2.2. Equipment Under Test (EUT)

The study compares two generations of wireless technology.

The WiFi 6 router model ASUS ROG Rapture GT-AXE11000 (ASUSTeK Computer Inc., Taipei, Taiwan—for both Wi-Fi routers) (Figure 1b) supporting the IEEE 802.11ax standard (limited to 160 MHz channels and 1024-QAM) was configured in a tri-band architecture, operating simultaneously in the 2.4 GHz band and two distinct segments of the 5 GHz spectrum (Lower 5 GHz and Upper 5 GHz). This configuration allowed for a comparison between high-capacity legacy systems. For WiFi 7 assessment, a router model ASUS ROG Rapture GT-BE19000WiFi (Figure 1c) was used, which introduces 320 MHz bandwidth, 4096-QAM, and Multi-Link Operation (MLO) for simultaneous triple-band data aggregation.

The mobile terminals consisted of two high-end WiFi 7 smartphones as clients: the Samsung Galaxy S25 (phone no. 1—Samsung Electronics Co., Ltd., Suwon, Republic of Korea) and Google Pixel 9 Pro XL (phone no. 2—Google LLC, Mountain View, CA, USA). These devices were selected for their distinct hardware implementations of the 802.11be standard, specifically their tri-band MIMO arrays and channel aggregation.

### 2.3. Measurement Instrumentation and Methodology

To capture the high-fidelity dynamics of WiFi 7 signals, the experimental setup relied on a passive metrological chain consisting of a PBS-1 E-field probe (Aaronia AG, Strickscheid, Germany) interfaced with a Rohde & Schwarz FSW-26 signal analyzer (Rohde & Schwarz GmbH & Co. KG, Munich, Germany) (with a maximum resolution bandwidth, RBW = 40 MHz, and a maximum analysis bandwidth (AnBW) = 512 MHz)—Figure 1. The decision to use a passive sensing configuration was deliberate; by bypassing active amplification stages, we ensured maximum linearity and eliminated the risk of intermodulation distortions or electronic noise artifacts. This integrity is paramount when analyzing the complex envelope fluctuations inherent to 4096-QAM modulations and MLO, where even minor hardware-induced compression can skew the statistical representation of signal peaks.

The primary challenge of a passive setup—inherent insertion loss—was mitigated by the high sensitivity of the FSW-26. By optimizing the analyzer’s Displayed Average Noise Level (DANL) to approximately −150 dBm/Hz, the system maintained a dynamic range wide enough to prevent stochastic thermal noise from masking the lower probability tails (10^−3^ to 10^−4^) of the CCDF. This sensitivity was especially critical given the 320 MHz AnBW used to encompass the full bandwidth of WiFi 7 channels. At such wide bandwidths, noise integration can typically elevate the noise floor, yet the optimized DANL ensured that the recorded PAPR remained an undistorted reflection of the actual signal physics.

Metrological traceability was maintained by integrating frequency-specific Antenna Factors (AF) for the 2.4, 5, and 6 GHz bands directly into the analyzer’s processing unit (transducer factor calibration). This allowed for the real-time conversion of received power (dBm) into absolute E-field strength (dBµV/m). Ultimately, the synergy between the isolation provided by the anechoic environment and the high-performance backend of the FSW-26 allowed us to capture the authentic electromagnetic footprint of the devices, accounting for both the modulation dynamics and the subtle scattering effects induced by the human operators.

### 2.4. Traffic Generation

To simulate realistic high-load scenarios, the network was saturated using iPerf3 (version iPerf 3.14) software. Controlled User Datagram Protocol (UDP) and Transmission Control Protocol (TCP) traffic streams were generated between the mobile terminals and a local server connected via a 10 GbE link to the routers. This setup allowed for the achievement of maximum achievable throughput (v), ensuring that the WiFi 7 features (such as MLO and 320 MHz bandwidth) were fully active during the EMF sensing process, thereby representing a “conservative-case” exposure scenario under functional data load.

### 2.5. Human-Centric Experimental Design

To isolate the electromagnetic footprint of the routers’ infrastructure, the test environment was organized into a polarized spatial configuration within the anechoic chamber. The routers GT-BE19000 (WiFi 7) and respectively the GT-AXE11000 (WiFi 6) were positioned so as to prevent the uplink emissions from the client devices (phones) from interfering with the router’s characterization, since the routers served as the primary sources of emission.

The E-field probe was placed in the immediate proximity of the router’s antenna array. This spatial separation ensured that the FSW-26 captured the downlink traffic and envelope fluctuations with high purity, focusing on the router’s output during high-load iPerf3 sessions. This rigorous geometry allowed for an authentic assessment of the router’s radiated field.

Distinct from conventional theoretical models, this study adopted a human-centric approach by involving three operators inside the anechoic chamber during the measurement sessions. The E-field probe was positioned at two strategic distances from the radiating routers to simulate real-life proximity:

- Near-field simulation (20 cm): representing a user holding a mobile device or sitting in immediate proximity to a router.
- Intermediate-field simulation (1 m): representing a standard office or home environment distance.

To ensure the capture of maximum exposure levels, a preliminary spatial scanning procedure (at 1 cm distance) was implemented to identify the most emissive antenna elements of the WiFi 6 and WiFi 7 routers. Since modern routers utilize complex internal antenna arrays and beamforming technologies, the physical origin of the strongest signal is not visually apparent.

a. *Antenna Mapping with the Near-Field Probe:*

Before the formal recording of data, the Aaronia PBS-1 near-field probe was used to perform a high-resolution surface scan of the router chassis. By moving the probe in a grid pattern while the router was under full iPerf3 traffic load, we identified the specific internal antenna elements responsible for the highest electromagnetic emissions in the 2.4 GHz, 5 GHz, and 6 GHz bands. This step was vital to ensure that the broadband E-field probes and the FSW input were subsequently aligned with the actual “hotspots” of the radiating source.

b. *Mobile Terminal Positioning (Beamforming Alignment):*

The positioning of the mobile terminals was also optimized based on real-time signal feedback. To simulate an intense exposure scenario, the smartphones were placed in the primary path of the router’s radiation lobes. We utilized the signal strength indicators and the FSW’s real-time spectrum analysis to verify that the mobile devices were positioned at the focal points where the beamforming gain was maximized. This alignment ensured that the MLO performance was peak-limited by the hardware’s capabilities and not by poor spatial orientation, thereby resulting in the highest possible E-field intensity (mV/m) at the simulated user’s position.

The operators were positioned to mimic typical usage postures, purposefully introducing the absorption, scattering, and reflection variables that occur in everyday wireless interactions. This design ensures that the captured CCDF statistics and field intensity levels reflect the actual impact of the human body on the WiFi 6 & 7 signal propagation and the resulting exposure footprint.

**Figure 2:**
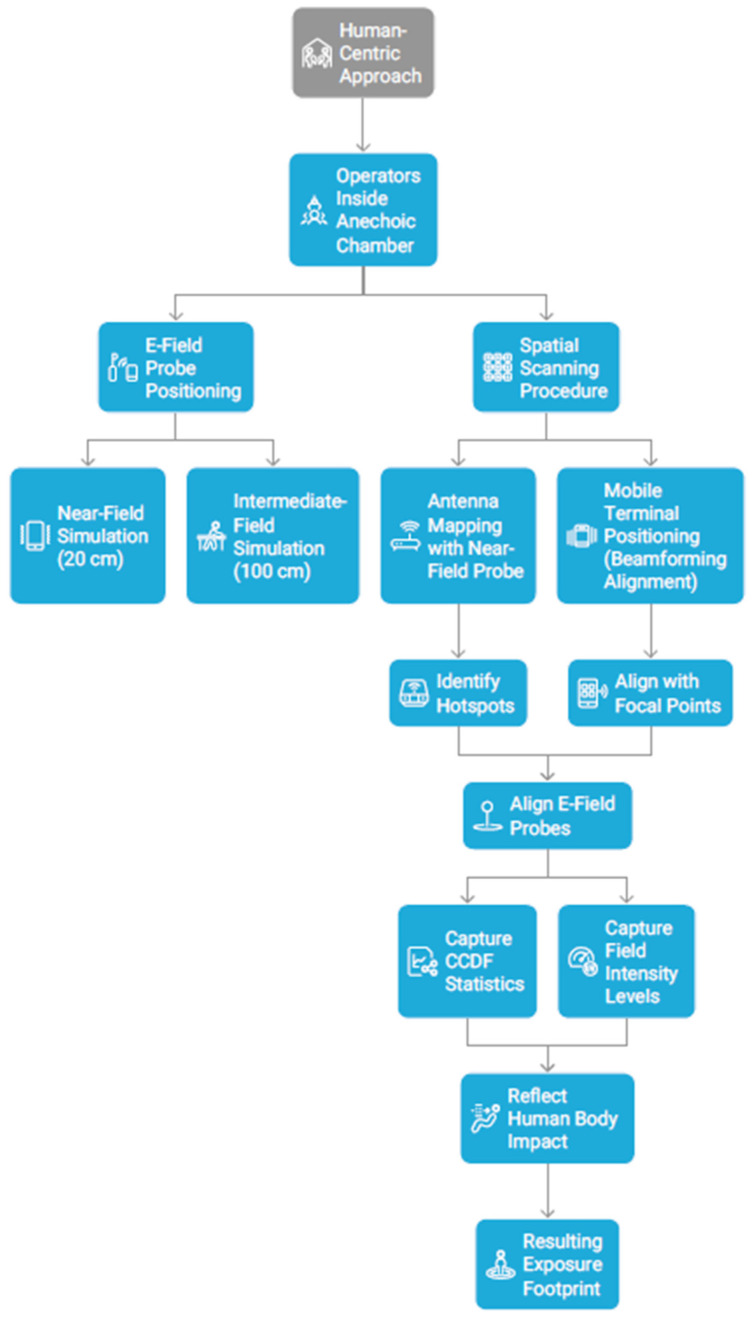
Workflow of human-centric full-stress exposure assessment to WiFi 6 and WiFi 7 router emissions.

The scheme of the methodological approach is presented in Figure 2.

## 3. Measurement Protocol

### 3.1. Scenario Definitions

To capture the full spectrum of the electromagnetic footprint, we defined three distinct operational scenarios. These scenarios were applied to both the WiFi 6 and WiFi 7 platforms, allowing for a direct comparison of how different generations manage energy emission under varying network demands.

*Scenario I: Idle State (Baseline):* The first scenario establishes the radio silence baseline. Here, the routers are powered on but no active data transfer is initiated by the client handsets. This stage is crucial for identifying the periodic beacon frames and background signaling. By comparing the Idle state of WiFi 6 and WiFi 7, we can determine the standby exposure floor and verify if the wider 320 MHz capability of the newer standard introduces any significant change in the Idle-state spectral density.

*Scenario II: Single-User, Full Traffic:* In this scenario, a single client (phone no. 1—Samsung Galaxy S25) is subjected to a heavy downlink load via iPerf3. For WiFi 6, this represents the saturation of a 160 MHz channel using 1024-QAM. For WiFi 7, we push the device to its 320 MHz limit with 4096-QAM modulation. This comparison isolates the impact of the new modulation scheme and increased bandwidth on the CCDF curve, highlighting how the peak dynamics shift when a single user consumes maximum resources.

*Scenario III: Multi-User, Full Traffic with MLO Active:* The final scenario represents the pinnacle of the WiFi 7 standard, MLO. While WiFi 6 is limited to single-band connectivity (switching between 5 GHz lower or 5 GHz upper band), Scenario III involves both handsets operating simultaneously to aggregate traffic across multiple bands. This allows us to measure the ‘superposition’ of electromagnetic fields. We focus on how MLO changes the exposure profile—not just by increasing throughput, but by creating a more complex, multi-band radiated field that significantly alters the statistical distribution of the peak E-field levels compared to the legacy single-link approach of WiFi 6.

### 3.2. Procedure of Expressing E-Field Level Across Different Frequency Bands and Scenarios

Since the Aaronia PBS-E1 is a monopolar probe, it captures the E-field strength primarily along a single axis. To determine the total E-field magnitude, a systematic three-axis measurement was implemented at each test point. The vectorial reconstruction followed a strict protocol: the *X*-axis was aligned parallel to the router’s main chassis, the *Y*-axis perpendicular to it in the horizontal plane, and the *Z*-axis oriented vertically. This ensured a full spatial capture of the E-field vector, regardless of the antenna polarization.

The probe was oriented along the three mutually orthogonal axes (*X*, *Y*, and *Z*), and the resultant field was reconstructed:

(1) $$E=\sqrt{E_{x}^{2}+E_{y}^{2}+E_{z}^{2}}$$

To minimize measurement uncertainty caused by human interference, the probe was mounted on a wooden tripod, allowing the operator to remain at a distance of at least 40 cm during data acquisition to prevent wave reflections or absorption.

a. *Idle Mode Analysis: use of Time Domain Power*

**Figure 3:**
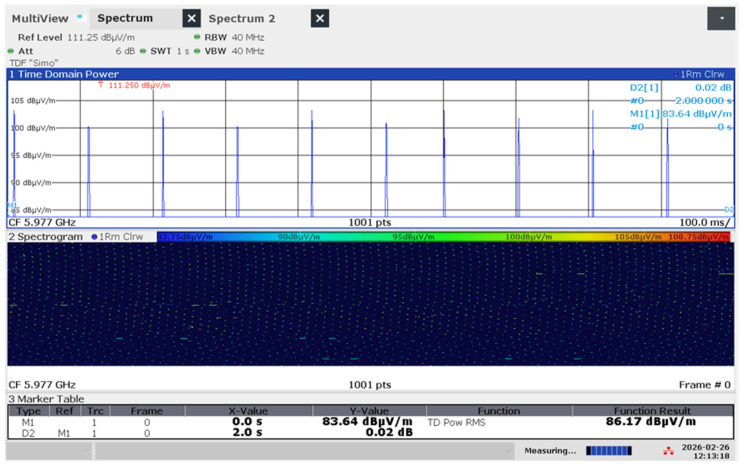
Idle state measurement with Time Domain Power of router emitting in WIFI 7 on 6 GHz band, emphasizing also the spectrogram.

To capture the short-duration, periodic bursts without the use of a hardware trigger, the analyzer was configured in Time Domain (Zero Span) mode using a Free Run acquisition. The measurement protocol was optimized with the following technical parameters: RBW = 40 MHz (the maximum capability of the analyzer, to ensure that the wideband nature of the WiFi beacons was captured with minimal amplitude distortion); sweep time (SWT) = 1 s (this duration is significantly longer than the standard WiFi beacon interval, ensuring that multiple emission bursts were captured within a single sweep, for a statistically representative sample of the Idle state); the Time Domain Power tool was employed to calculate the mean power over the entire 1 s trace (Figure 3); this provides a stable “Time-Average” E-field strength, which is the most relevant metric for assessing human exposure; detector type = RMS (to integrate the power, focusing on the average thermal energy of the field rather than instantaneous peaks); Trace was averaged over a sweep count = 10.

b. *Active Traffic and MLO: use of Channel Power*

**Figure 4:**
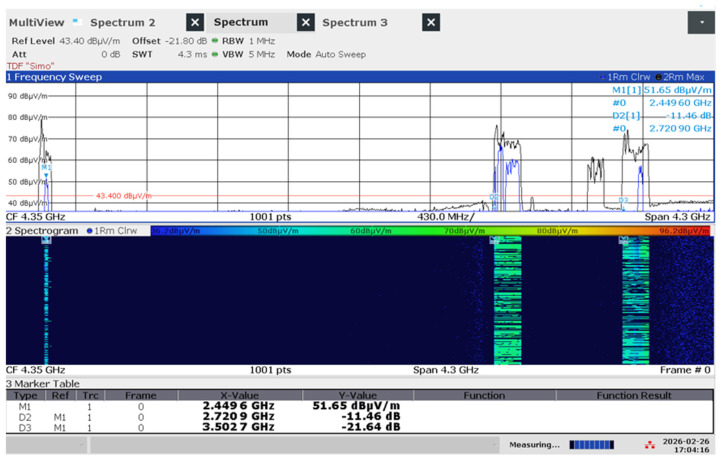
Spectrum and spectrogram of the MLO emissions in WIFi 7 (2.4 GHz, 5 GHz and 6 GHz bands).

**Figure 5:**
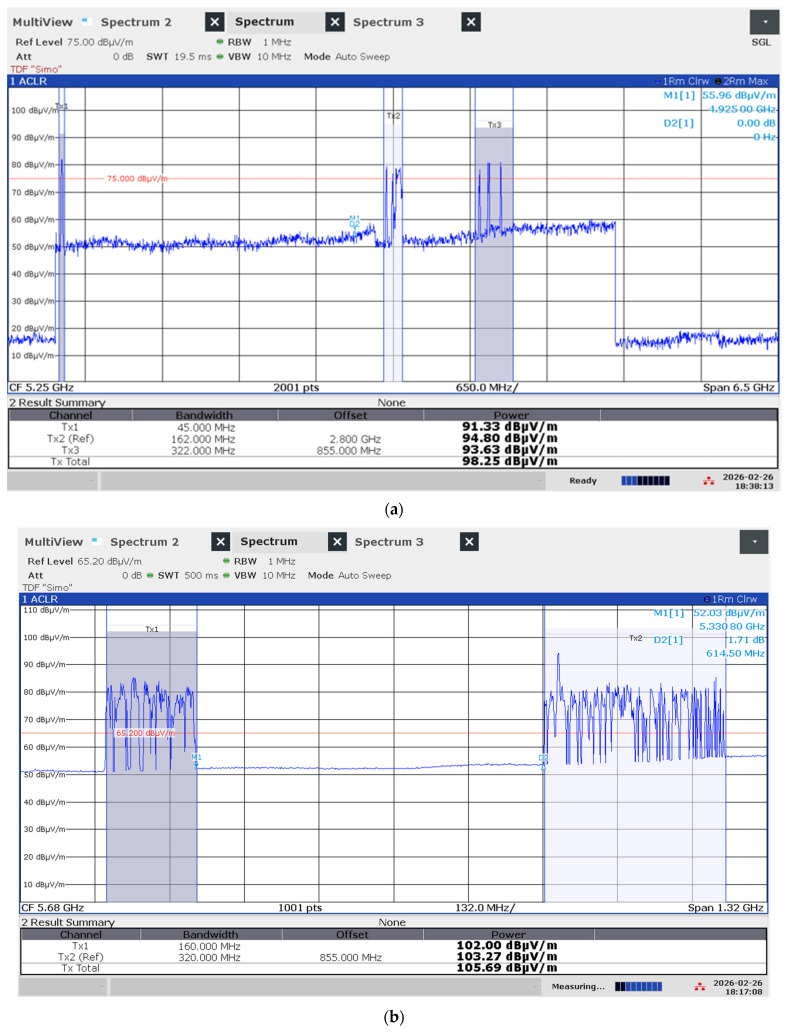
Two examples of Channel Power method applied to measure WiFi 7—MLO case: (**a**) tri-band MLO with figured E-field strength in each band and total field strength in the aggregated emission; (**b**) dual-band high MLO case.

For scenarios involving active data transfer (iPerf3 UDP stress tests), the Channel Power function was employed. The integration bandwidth was set to 160 MHz for WiFi 6 and expanded to 320 MHz for WiFi 7 to encompass the full spectral occupancy. In MLO scenarios, where the router transmits simultaneously across multiple bands (Figure 4), a specialized split-channel power function was utilized, named multi-carrier channel power, to compute each channel’s power in the 2.4 GHz, 5 GHz, and 6 GHz segments and the total power (Figure 5a,b). The total exposure was calculated as the cumulative sum of the power densities across all active links, following the same three-axis vectorial reconstruction protocol to ensure a conservative and accurate assessment of the maximum field intensity. The settings of the analyzer were: Detector = RMS; Trace mode = Average (10 sweeps); sweep time = 100–200 ms; the Integration Bandwidth (IBW) was precisely matched to the nominal channel bandwidth (80, 160, or 320 MHz) to ensure that the total power of the multi-carrier signal was fully captured without spectral truncation.

### 3.3. Statistical Analysis (CCDF)

To correctly understand the exposure profile of a WiFi 7 signal, traditional average power measurements are often insufficient. Because standard 802.11be relies on extremely dense modulations (4096-QAM) and wideband multi-link operations, the resulting signals are characterized by rapid, high-amplitude bursts that are lost in time-averaged metrics. We therefore employed CCDF analysis as our primary statistical tool to capture these transient dynamics. The fundamental reason for choosing CCDF lies in its ability to quantify PAPR. In a high-load iPerf3 scenario, the signal envelope is anything but constant; it is a stochastic process where instantaneous power peaks can significantly exceed the average level. By using CCDF, we can precisely determine the percentage of time the signal spends at or above a specific power threshold.

Graphically, a CCDF curve displays the probability that the instantaneous signal power will exceed the average power by a specific number of dB—this exceeding is known as the PAPR (Crest Factor—as analyzer denotes). During the measurement process, the analyzer performs high-speed statistical processing of millions of signal samples in real time. On the instrument’s display, the vertical axis represents the probability (ranging from 100% down to 0.0001%), while the horizontal axis indicates the power level above the average (dB).

To stimulate the peak emission capabilities of the WiFi 7 interface, we utilized iPerf3 as a network stress-test tool. The methodology was designed to transition the router from a passive Idle state to an Extremely High Throughput (EHT) condition. Unlike standard web browsing, which generates sporadic and unpredictable traffic, iPerf3 allowed us to saturate the 320 MHz channel by generating multiple parallel TCP and UDP streams. This saturation is vital for our statistical analysis because it forces the router to utilize its maximum modulation coding scheme (4096-QAM) and activates the full potential of MLO across the 5 GHz and 6 GHz bands.

**Figure 6:**
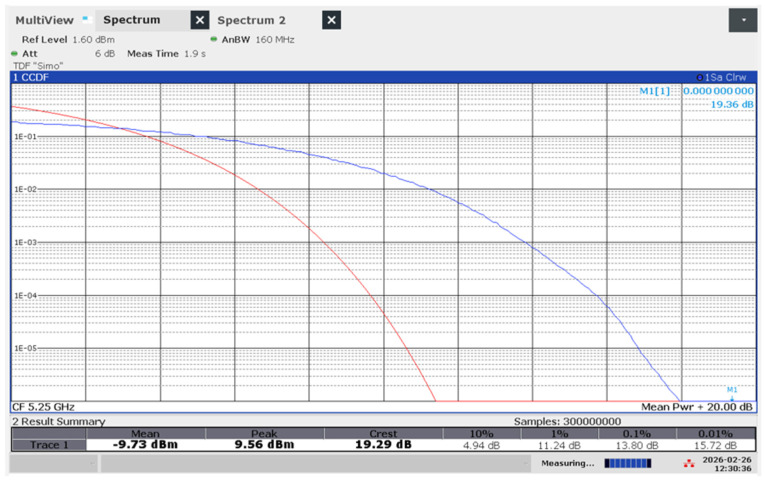
Example of CCDF trace in a 160 MHz bandwidth at 5 GHz in WIFi 7 with single phone connected. Red trace—the theoretical Gaussian reference (AWGN), serving as a baseline for a signal with a normal distribution; Blue trace—the actual measured WiFi 7 signal, illustrating PAPR.

From a measurement perspective, the impact of this traffic load is immediately visible on the FSW-26 analyzer’s CCDF trace. As the iPerf3 session initiates, the statistical fingerprint of the signal shifts: the curve moves to the right, indicating a higher PAPR (Figure 6). This shift represents the moment when the complex WiFi 7 wave-packets—carrying dense data payloads—begin to dominate the electromagnetic environment. By correlating the throughput reported by iPerf3 with the probability levels on the CCDF curve, we can establish a direct link between network performance and the intensity of the transient peaks to which a human operator is exposed.

## 4. Results and Discussion

### 4.1. E-Field Strengths in Idle State

**Figure 7:**
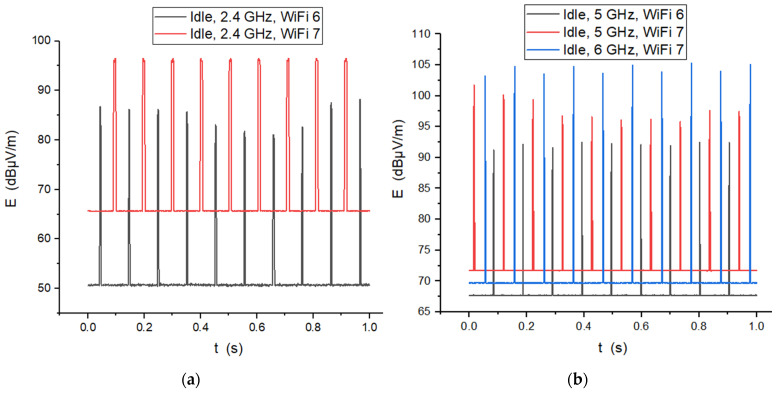
Time domain field strengths; comparative view between the two standards in the case of routers emitting at 20 cm distance, in cases: (**a**) 2.4 GHz Idle state; (**b**) 5 GHz and 6 GHz Idle state.

A compelling detail emerging from the 2.4 GHz Idle recordings (Figure 7a) is the distinct upward shift in the baseline E-field level for WiFi 7 compared to WiFi 6. This elevated baseline suggests that WiFi 7 hardware maintains a higher state of operational readiness—likely due to the more complex biasing requirements of EHT radio chains and the overhead of maintaining multi-link synchronization. The beacon frames in WiFi 7 appear slightly broader in the time domain. This duration increase is consistent with the expanded management payload mandated by the 802.11be standard, which must broadcast additional coordination parameters for the 5 GHz and 6 GHz bands even when idling on the 2.4 GHz interface. Essentially, while WiFi 7 is more efficient during data bursts, its quiet state carries a slightly heavier electromagnetic signature than its predecessor does.

Figure 7b presents the beacons profile in time for the 5 and 6 GHz bands. The most striking observation is found in the 6 GHz WiFi 7 signal (blue trace), which exhibits the highest peak-to-baseline amplitude. This suggests a high-intensity pulsing strategy specifically designed to maintain network discoverability in the 6 GHz band, despite its inherent propagation challenges. The 5 GHz WiFi 7 signal (red trace) follows with a moderate amplitude but sits on a significantly higher baseline, indicating a more persistent electromagnetic floor. Finally, the 5 GHz WiFi 6 signal (black trace) shows the lowest overall amplitude and the most discrete pulsing. This progression clearly demonstrates that WiFi 7 (802.11be) introduces not only a higher background energy state but also significantly more powerful management bursts compared to its predecessor, particularly as we move into the 6 GHz spectrum.

**Figure 8:**
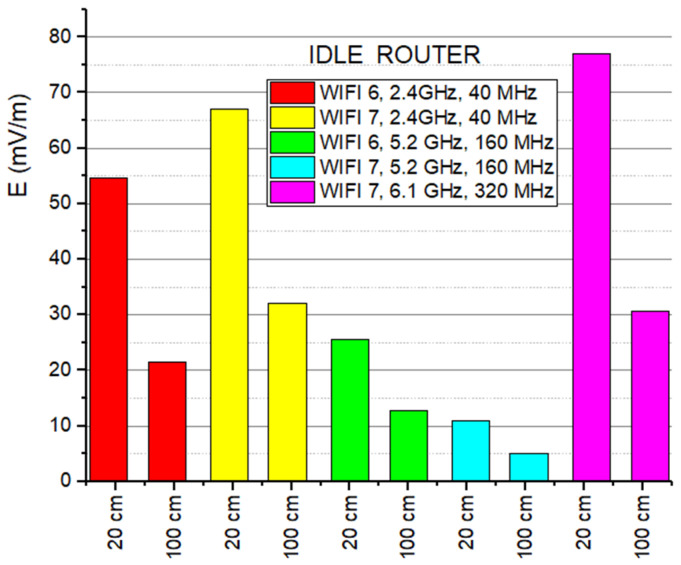
Comparative E-field strength values in Idle mode, at the two distances.

Comparative measurements of the emitted field level in the Idle state reveal a fundamental divergence between the two standards, suggesting that WiFi 7 manages control signaling differently than its predecessor (Figure 8). In the 2.4 GHz band, WiFi 7 introduces a higher exposure floor, reaching 66.92 mV/m at 20 cm compared to the 54.67 mV/m generated by WiFi 6. This increase is due to a denser management overhead required to maintain synchronization in increasingly crowded spectral environments. Interestingly, the situation reverses in the 5 GHz band, where WiFi 7 becomes significantly “quieter,” reducing field intensity to just 10.98 mV/m against the 25.62 mV/m recorded for WiFi 6 at 20 cm away. Meanwhile, the newly introduced 6 GHz band raises the electromagnetic baseline to 76.96 mV/m, a direct consequence of the massive 320 MHz bandwidth used for network discovery.

When observing the transition from 20 cm to 100 cm, the E-field levels drop significantly across all bands (Figure 8), yet the attenuation does not strictly follow the ideal 1/d^2^ theoretical curve. The WiFi 7—5 GHz signal aligns most closely with theoretical expectations, exhibiting a sharp 53.9% reduction in field intensity as distance increases. In contrast, the WiFi 6 at 2.4 GHz emission deviates most significantly from the theory, showing a more conservative 60.7% decrease despite the five-fold increase in distance. This disparity occurs because the 5 GHz band’s shorter wavelength behaves more like a directional beam that dissipates rapidly, whereas the 2.4 GHz band’s longer wavelength is more resilient to path loss and prone to environmental persistence. Furthermore, the advanced MIMO antenna arrays in these devices create complex constructive interference patterns that can sustain higher field levels at 100 cm than a simple point-source model would predict.

### 4.2. E-Field Strength in Forced Full Traffic, with One or Two Phones Connected to the Router

**Figure 9:**
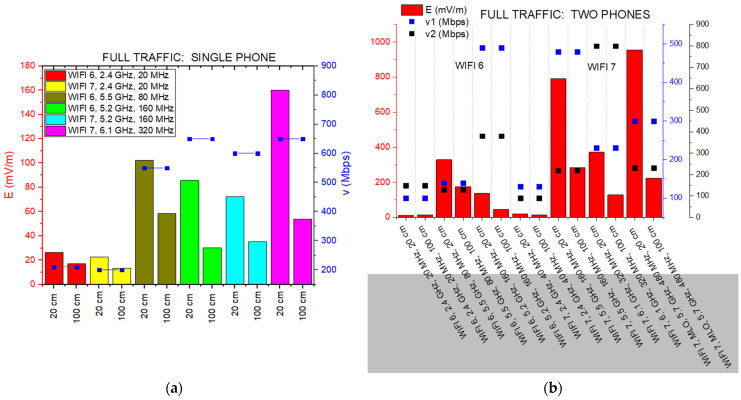
(**a**) E-field strength in iPref 3 full traffic conditions and respective throughputs (v1 & v2 in Mbps), at two distances from the router, in the cases: (**a**) with one phone connected; (**b**) with two phones connected.

When we transition from a resting state to active data transfer with one phone connected, the relationship between the throughput v (Mbps) and signal intensity becomes much more dynamic (Figure 9a). In the 5 GHz band, WiFi 7 proves to be remarkably efficient; it sustains a transfer rate of 600 Mbps with a field strength of 72.49 mV/m, whereas WiFi 6 generates a slightly higher intensity of 85.67 mV/m to reach a similar speed of 650 Mbps. This indicates that the newer standard is capable of handling heavy traffic more smoothly, essentially delivering more data without a corresponding rise in signal presence. However, the 6 GHz band introduced with WiFi 7—while offering a peak speed of 650 Mbps—shows a much higher initial signal level of 160.14 mV/m at 20 cm distance. In the more common 2.4 GHz band, both standards perform similarly, though WiFi 7 maintains a slightly higher footprint of 22.49 mV/m compared to the 16.47 mV/m seen with WiFi 6.

The way these signals distribute themselves over distance also reflects the unique setup of the experiment, including the presence of three human operators. The 6 GHz signal follows a predictable path, with its intensity decreasing by 66.5% at a distance of 1 m. In contrast, the 2.4 GHz signal from the WiFi 7 router remains much more stable, dropping by only 41.4% at the same mark. This stability, which deviates from ideal theory, is likely enhanced by MIMO antenna arrays and the scattering effects caused by the human presence in the chamber, which prevents a simple linear decay. Ultimately, while the 6 GHz band reaches higher levels, WiFi 7 operates with impressive balance in the established 5 GHz spectrum, even in a complex, multi-occupant environment.

As illustrated in Figure 9b, the full traffic stress test involving two simultaneous smartphone clients (with different speeds, noted by v1—blue marks; v2—black marks) reveals several critical trends regarding E-field intensity and aggregate throughput.

Under WiFi 6 conditions, we observe a relatively high E-field penalty, particularly in the 5 GHz band. For instance, at a 20 cm distance with an 80 MHz bandwidth, the field intensity reaches significant levels (~330 mV/m) while providing asymmetrical throughput for the two devices. Interestingly, while increasing the bandwidth to 160 MHz in the 5.2 GHz band drops the E-field levels, the throughput remains capped, suggesting that the hardware works harder (with higher emission spikes) to maintain stability in narrower or more congested spectral segments.

The introduction of WiFi 7 (802.11be) clearly amplifies the performance envelope, but not without a corresponding increase in the electromagnetic footprint during traditional single-link operations. The 5.5 GHz (160 MHz) and 6.1 GHz (320 MHz) configurations show the highest E-field peaks in the study, exceeding 800–900 mV/m at 20 cm. This is likely due to the aggressive 4096-QAM modulation and the increased radio activity required to sustain high-velocity data streams for multiple users simultaneously.

The most compelling results, however, emerge from the MLO scenario at an aggregate bandwidth of 480 MHz (achieved through the simultaneous aggregation of a 160 MHz channel in the 5 GHz band and a 320 MHz channel in the 6 GHz band). This configuration represents the pinnacle of the study’s performance metrics, albeit with a complex electromagnetic profile.

At a close proximity of 20 cm, the MLO setup generates the absolute peak E-field intensity of the entire dataset, reaching approximately 960 mV/m. This is a direct physical consequence of the router’s radio front-end driving maximum power across an aggregated 480 MHz pipe to satisfy the heavy data demands of both clients simultaneously. The behavior of the MLO signal at a 100 cm distance reveals a nuanced propagation evolution. While the E-field drops significantly to ~220 mV/m, it does not follow a uniform hierarchy compared to single-link WiFi 7 modes. Specifically, at this 1 m position, the MLO exposure remains lower than the 5.5 GHz (160 MHz) single-link (~280 mV/m), yet stays higher than the 6.1 GHz (320 MHz) link (~130 mV/m). This suggests that while the 6 GHz band suffers from rapid atmospheric and structural attenuation, the 5.7 GHz component within the MLO aggregate sustains the field more effectively over distance.

Crucially, the throughput data (v1, v2) provides the necessary context for these emission levels. In the MLO results, we observe a distinct stabilization of the data streams compared to the more erratic behavior of legacy standards. While the throughput for the first device (v1) reaches approximately 300 Mbps, the second device maintains a consistent, albeit slightly lower, rate (v2) of approximately 250 Mbps. Although these values are not identical, MLO demonstrates a superior ability to sustain dual-client traffic under full traffic stress, avoiding the total throughput collapse or extreme asymmetry seen in the 5 GHz WiFi 6 scenarios. This suggests that the high E-field observed at 20 cm (~960 mV/m) is the price for maintaining this multi-device connectivity, ensuring that both users retain functional high-speed access even under maximum network load.

From another perspective, E-field attenuation with distance from the router observed in Figure 9b is the result of a complex interplay between the operational frequency and the high-order modulation schemes.

The WiFi 7 MLO (480 MHz) case exhibits the most aggressive spatial decay in the entire study, with the E-field intensity collapsing by approximately 76.6%. This is followed by the WiFi 7 at 6.1 GHz (320 MHz) and 5.5 GHz (160 MHz) configurations, which show reductions of 65.5% and 63.9%, respectively. This phenomenon is strictly governed by the physics of higher-frequency bands and wider integration bandwidths. The shift to these expanded spectral segments in WiFi 7 inherently ensures that the E-field dissipates much faster across the 1 m span than in legacy standards. Conversely, the WiFi 6 (2.4 GHz) signals maintain a nearly static footprint, illustrating the high persistence of longer wavelengths over distance.

The transition from 1024-QAM (WiFi 6) to 4096-QAM (WiFi 7) imposes extreme requirements on signal integrity. To achieve the very low Error Vector Magnitude (EVM) necessary for 4096-QAM, the router must deliver a very high Signal-to-Noise Ratio (SNR). In the near-field (20 cm), this necessitates the significant power density observed in the MLO peak and the 5.5 GHz WiFi 7 link. This clean and intense signal is required to distinguish between the 4096 tightly packed constellation points, ensuring data symbols are not lost to noise. As the distance increases to 100 cm, the sharp drop in E-field across all WiFi 7 high-bandwidth modes makes maintaining such a low EVM increasingly difficult. However, the throughput data (v1, v2) reveals a remarkable stability: despite the 76.6% field reduction in MLO, the data rates remain constant at 300 Mbps and 230 Mbps respectively. This suggests that the system is highly optimized, likely employing sophisticated beamforming or the inherent redundancy of multi-link aggregation to compensate for the field collapse.

This confirms a “self-limiting” exposure profile: the more advanced the modulation and the higher the frequency, the more the high-intensity exposure is confined to the immediate vicinity of the transmitter. WiFi 7 effectively trades high near-field intensity for a rapid far-field safety buffer, providing superior data capacity without a proportional increase in long-range electromagnetic footprint.

### 4.3. Exposure Burden per Throughput

The metric exposure burden per throughput expresses the amount of electromagnetic power density (S, in pW/cm^2^) associated with each unit of data throughput, meaning that higher values correspond to a higher exposure burden per transmitted Mbps, while lower values indicate more exposure-efficient data transmission.

(a) *Traffic with single phone:*

**Figure 10:**
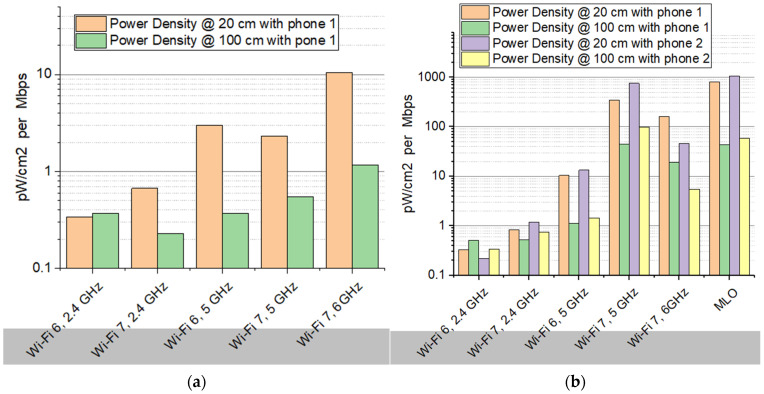
Exposure efficiency expressed as normalized power density to the throughput in the cases: (**a**) a single phone connected; (**b**) two phones connected to the router.

The first important observation of this case, shown in Figure 10a, is the consistent effect of distance. For all Wi-Fi configurations, the exposure burden measured at 100 cm is significantly lower than at 20 cm. In most cases, the reduction approaches approximately one order of magnitude, highlighting the strong influence that spatial separation from the access point has on exposure levels.

When examining the different wireless technologies, a general increase in exposure burden can be observed as the system moves toward higher-frequency bands and more advanced Wi-Fi generations. The lowest values appear for Wi-Fi 6 operating at 2.4 GHz, where the exposure burden remains below approximately 0.4 pW/cm^2^ per Mbps even at a short distance of 20 cm. This indicates that relatively little electromagnetic power density is associated with each transmitted unit of data in this configuration. For Wi-Fi 7 at 2.4 GHz, the exposure burden increases slightly at 20 cm but decreases somewhat at 100 cm compared with Wi-Fi 6 in the same band. This suggests that improvements in the protocol and transmission mechanisms may allow somewhat more efficient data delivery at larger distances under the tested conditions.

A more pronounced increase in exposure burden is visible when moving to the 5 GHz band. Both Wi-Fi 6 and Wi-Fi 7 at 5 GHz exhibit higher values compared with the 2.4 GHz configurations, especially at 20 cm. This indicates that higher-frequency operation, while typically enabling higher throughput, may also be associated with a larger electromagnetic exposure burden per transmitted Mbps in this experimental setup.

The highest exposure burden in the single-phone scenario is observed for Wi-Fi 7 operating at 6 GHz. At 20 cm, the exposure burden approaches roughly 10 pW/cm^2^ per Mbps, which is substantially higher than the values observed for the other configurations. Even at 100 cm, this configuration still exhibits the largest exposure burden among the tested cases.

While the 6 GHz band offers significant throughput advantages, its global regulatory status varies, with different regions (e.g., FCC vs. CEPT) adopting varying power limits and channel availability, which directly influences the observed exposure burden in real-world deployments.

(b) *Traffic with two phones:*

A first observation in this case (Figure 10b) is that the general trends identified in the single-device scenario remain visible, but the magnitude of the exposure burden increases substantially when two devices are active at the same time. This suggests that simultaneous multi-device communication may lead to a higher electromagnetic exposure burden per transmitted data unit under the tested conditions.

Distance continues to play a significant role. For all Wi-Fi configurations and for both phones, the exposure burden measured at 100 cm is consistently lower than at 20 cm, confirming the expected reduction in electromagnetic exposure with increasing distance from the router. However, the difference between the two distances becomes even more pronounced in the two-device scenario, particularly for the higher-frequency configurations.

Compared with the single-phone case, the most notable change is the strong increase in exposure burden for the more advanced Wi-Fi configurations, especially those associated with Wi-Fi 7 and MLO. At a distance of 20 cm, the exposure burden rises to values in the hundreds or even around one thousand pW/cm^2^ per Mbps, which is significantly higher than the levels observed when only one phone was connected. This indicates that the electromagnetic exposure burden per unit of delivered data increases considerably when multiple devices simultaneously share the wireless channel.

Another relevant aspect is the difference between the two phones. In most configurations, Phone 2 (Google Pixel 9 Pro XL) shows higher exposure burden values than Phone 1 (Samsung Galaxy S25), particularly at short distance and for the more advanced Wi-Fi modes. This suggests that device-specific factors such as antenna design, radio front-end implementation, or power control algorithms may influence the interaction with the access point, leading to measurable differences in exposure burden.

Looking at the frequency bands, the 2.4 GHz configurations still exhibit the lowest exposure burdens, even when two devices are connected. In contrast, Wi-Fi 7 at 5 GHz and 6 GHz shows a dramatic increase in exposure burden, especially at 20 cm. The highest values appear for MLO, where multiple frequency links are used simultaneously to improve throughput and latency. While this mechanism enhances performance, the results indicate that it may also lead to the largest exposure burden per transmitted Mbps among the tested configurations.

When comparing the two experimental scenarios, several key differences emerge: Exposure burden per throughput is significantly higher when two phones are connected simultaneously than in the single-device case. The increase is particularly pronounced for advanced Wi-Fi technologies such as Wi-Fi 7 and MLO. Device-specific differences become more visible in the multi-device scenario. Distance continues to reduce exposure burden substantially in all configurations.

Overall, the results suggest that network load and the number of simultaneously connected devices can play an important role in the electromagnetic exposure burden associated with wireless communication. While advanced Wi-Fi technologies enable higher performance and improved connectivity, the measurements indicate that their exposure burden per transmitted data unit may increase considerably when multiple devices operate concurrently.

### 4.4. The Constellation Challenge: The 4096-QAM Instability Due to the Human-Centric Setup

Despite the controlled conditions of the anechoic chamber, capturing stable constellation diagrams for the 4096-QAM modulation specific to WiFi 7 proved to be an exceptional challenge, as mentioned in Section 4.2. The extreme density of the symbol constellation in 802.11be requires a very high SNR and a strictly low EVM, typically below −38 dB.

Our observations suggest that the human-centric approach—specifically the physical presence of operators within the chamber—introduced subtle multi-path fading and signal absorption. While these environmental perturbations might be negligible for lower-order modulations like those in WiFi 6, they were sufficient to cause the 4096-QAM constellation to collapse into a cloud-like distribution. Despite the observed dispersion in the constellation points caused by human-induced scattering, the iPerf3 throughput remained remarkably stable. This suggests that the router’s beamforming algorithms and adaptive modulation coding schemes effectively compensated for the physical perturbations, prioritizing link reliability over theoretical peak speeds.

**Figure 11:**
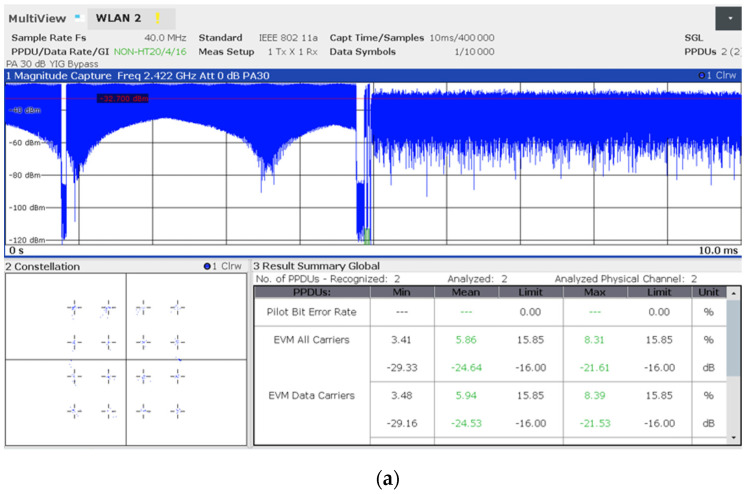

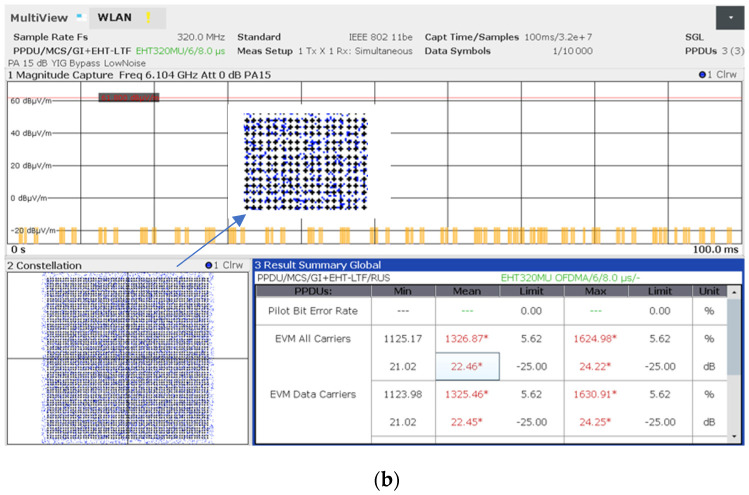
Display of the FSW analyzer showing an example of: (**a**) 16-QAM constellation measurement (WiFi 6); (**b**) 4096-QAM constellation (WiFi 7) measurement (with a zoom inset included). * High EVM values indicating a signal integrity failure and an insufficient SNR value for a successful demodulation.

Here we exemplify the constellation measurements obtained with 16-QAM (Figure 11a) and with 4096-QAM (Figure 11b) in average conditions of human-centric control in the anechoic chamber.

Consequently, this study shifted its focus toward CCDF analysis, which provides a more robust statistical characterization of power dynamics and PAPR under real-world human-centric exposure scenarios.

### 4.5. Statistical Power Dynamics and CCDF Analysis

The power characteristics of WiFi 6 and WiFi 7 signals were evaluated through Mean Power, Peak Power, and PAPR measurements, supplemented by a detailed CCDF analysis, at 1 m distance from the routers. The results are summarized in Table 1. It should be noted that data for the WiFi 6 ‘upper band’ in dual-phone scenarios is unavailable due to hardware synchronization limitations during simultaneous traffic stress. However, this does not compromise the study’s baseline, as the ‘lower band’ results provide a sufficiently representative characterization of legacy 802.11ax exposure dynamics for comparative purposes.

In single-terminal configurations, WiFi 6 is characterized by relatively low mean power levels but exhibits significant stochastic instability, particularly in the 5 GHz upper band where peak power goes to 21.15 dBm. This indicates a waveform with high PAPR (26.01 dB), subjecting the user to intense, albeit brief, electromagnetic transients. Conversely, WiFi 7 demonstrates superior power envelope control in its 6 GHz allocation; despite maintaining a consistent mean power, its peak levels are suppressed to 9.56 dBm. From a dosimetric and signal-characterization perspective, WiFi 7 transitions the exposure profile from high-intensity sporadic pulses toward a more linearized peak distribution. This transition is noteworthy because the temporal distribution of the energy (PAPR) changes, even though the total exposure remains well below established safety limits.

The transition to a dual-terminal environment highlights a fundamental divergence in protocol behavior. While WiFi 6 power metrics remain conservative or show instability in higher bands, WiFi 7 aggressively scales its output to sustain spatial multiplexing. In the 5 GHz middle band, the mean power shifts into positive range (+5.77 dBm), accompanied by the highest peak power recorded in this study (23.63 dBm). This suggests that under high-load conditions, WiFi 7 significantly increases the ambient power flux density compared to its predecessor, reflecting the energetic cost of its increased spectral efficiency.

Based on the extracted metrics from Table 1 it results that the WiFi 7—5 GHz (Middle Band) with two phones configuration represents the scenario with the highest recorded exposure levels. This determination is based on a convergence of three critical dosimetric factors: (a) maximum mean power (+5.77 dBm)—this is the primary determinant for time-averaged Specific Absorption Rate (SAR) of energy deposition and long-term dielectric heating of biological tissues; (b) absolute peak power (23.63 dBm)—this represents the highest instantaneous E-field strength incident upon the user, a key metric for evaluating non-thermal biological interactions; (c) absorption depth: unlike the 6 GHz band, where energy deposition is largely restricted to the cutaneous layers, the 5 GHz frequency possesses a greater penetration depth into underlying subcutaneous tissues. When coupled with the highest recorded energy density, this frequency–power combination constitutes the maximum biological load within the tested parameters. At these higher frequencies (5–6 GHz), the electromagnetic energy is primarily absorbed within the superficial skin layers. It is important to note that while our recorded levels are well below the ICNIRP basic restrictions, the localized peak intensities observed in WiFi 7 MLO modes approach the operational boundaries where thermal effects dominate the interaction mechanism, rather than the bulk-heating effects seen at lower frequencies.

The 0.01% CCDF threshold is a critical metric for understanding the persistence of high-power transients. In the identified conservative-case scenario (WiFi 7, two phones, 5 GHz band), the 0.01% value is 14.30 dB, while the absolute PAPR is 17.87 dB. The narrow delta of 3.57 dB between the statistical tail and the absolute peak suggests a very dense power envelope; the signal stays near its maximum intensity for a significant portion of the transmission time.

In contrast, looking at the WiFi 6, one phone connected, upper 5 GHz band (often cited for its high peaks), we see a 0.01% value of 21.98 dB and a PAPR of 26.01 dB. This larger gap of 4.03 dB, combined with much higher absolute values, characterizes a signal defined by extreme, but extremely rare spikes. While WiFi 6 hits higher instantaneous peaks in this band, WiFi 7’s energy is more “concentrated” near its peaks, leading to a more continuous electromagnetic load during active data transfer.

A significant methodological contribution of this work is the application of the 0.01% CCDF threshold. We propose that this statistical metric should serve as a model for future EMF monitoring of wideband wireless systems. Unlike traditional time-averaging, the 0.01% CCDF effectively captures the ‘worst-case’ peak dynamics of stochastic, high-bandwidth signals like WiFi 7, providing a more rigorous and repeatable framework for regulatory compliance in complex, human-centric environments.

**Figure 12:**
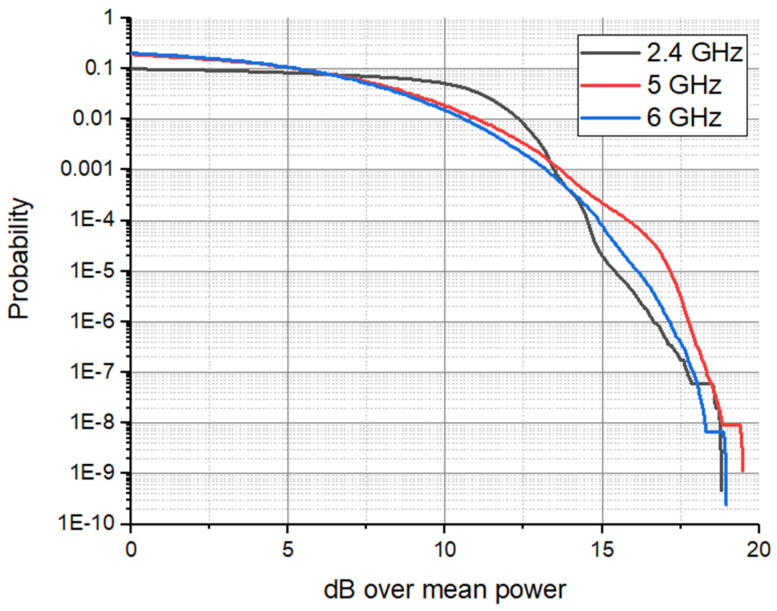
Superimposing of three CCDF curves when a single phone was connected to the Wi-Fi 7 router.

The experimental CCDF curves for the single-phone WiFi 7 configuration (Figure 12) demonstrate a high degree of convergence with the tabulated statistical metrics. While the 6 GHz band exhibits slightly higher power levels at the 0.01 probability threshold, a crossover occurs in the extreme tail (<0.0001), where the 5 GHz band (Middle Band) shows superior persistence. Specifically, the 5 GHz emission profile maintains a shallower decay slope as it approaches the measured PAPR of 19.44 dB. This suggests that even in single-device scenarios, the 5 GHz allocation subjects the biological target to more frequent high-intensity transients compared to the 2.4 GHz and 6 GHz counterparts.

## 5. Conclusions

This study provides a comprehensive metrological evaluation of the transition to IEEE 802.11be, emphasizing how WiFi 7’s core innovations redefine the EMF exposure landscape. Our experimental results demonstrate that while the integration of 320 MHz bandwidth and 4096-QAM modulation leads to higher instantaneous peak E-fields (reaching 955 mV/m in the near-field), it simultaneously optimizes the exposure burden per unit of throughput. A major finding is the superior ‘exposure efficiency’ of WiFi 7, where the Multi-Link Operation allows for a more balanced distribution of energy across bands, effectively reducing the temporal duration of high-intensity radiation compared to legacy standards.

The research highlights a critical spatial dynamic: WiFi 7 exhibits a rapid electromagnetic power decay (76.6% at 1 m from the router), ensuring that peak exposures are highly localized and dissipate much faster than in WiFi 6. Furthermore, our human-centric setup revealed that while physical presence induces ‘constellation collapse’ in 4096-QAM, the system’s beamforming and adaptive modulation maintain link stability, though at a measurable exposure penalty during multi-client synchronization. We also identified a significant exposure asymmetry between different hardware implementations (Phone A vs. Phone B), suggesting that device-specific RF front-end design remains a decisive factor in real-world dosimetry.

Methodologically, this work validates the 0.01% CCDF threshold as a more accurate and reproducible metric for characterizing wideband, stochastic signals than traditional time-averaging. In conclusion, WiFi 7 offers a significant technological leap by trading localized peak intensities for enhanced spectral efficiency and a lower far-field exposure profile. These findings provide a robust empirical foundation for future EMF compliance protocols and the development of exposure-aware wireless network planning.

## Figures and Tables

**Table 1:** Comprehensive Statistical Power Metrics and CCDF Data for WiFi 6 and WiFi 7.

Scenario	Mean Power	Peak Power	PAPR	10%	1%	0.10%	0.01%
	(dBm)	(dBm)	(dB)	(dB)	(dB)	(dB)	(dB)
WIFI 6, 1 phone, lower band	−19	4.47	23.46	0	13.17	17.52	19.75
WIFI 6, 1 phone, middle band	−10.4	10.27	20.67	4.5	12.33	14.91	16.85
WIFI 6, 1 phone, upper band	−4.87	21.15	26.01	0	15.05	20.13	21.98
WIFI 6, 2 phones, lower band	−22.8	−0.12	22.68	0	12.93	16.21	18.36
WIFI 6, 2 phones, middle band	−7.99	11.93	19.91	5.54	11.19	13.4	15.11
WIFI 6, 2 phones, upper band	— **	— **	— **	— **	— **	— **	— **
WIFI 7, 1 phone, lower band	−16.86	0.76	17.61	0	12.58	13.52	14.02
WIFI 7, 1 phone, middle band	−8.57	10.87	19.44	5.38	10.96	13.46	15.52
WIFI 7, 1 phone, upper band	−9.73	9.56	19.29	4.94	11.24	13.8	15.72
WIFI 7, 2 phones, lower band	−12.89	5.31	18.19	3.58	12.02	12.72	13.72
WIFI 7, 2 phones, middle band	5.77	23.63	17.87	5.32	10.4	12.98	14.30
WIFI 7, 2 phones, upper band	0.14	18.64	18.5	5.02	9.3	11.64	13.48

** Data not available due to hardware synchronization constraints.

## Data Availability

The data presented in this study are available on request from the corresponding author due to privacy reasons.

## References

[1] López-Pérez D., García-Rodríguez A., Galati-Giordano L., Kasslin M., Doppler K. (2019). IEEE 802.11be Extremely High Throughput: The Next Generation of Wi-Fi Technology Beyond 802.11ax. IEEE Commun. Mag..

[2] Khorov E., Levitsky I., Akyildiz I.F. (2020). Current Status and Open Problems of IEEE 802.11be, the Future Wi-Fi 7. IEEE Access.

[3] Zhang S., Yu L., Cheng Y. (2024). An Introduction to Key Technologies of Wi-Fi 7. Int. J. Front. Eng. Technol..

[4] Raventós Á., Bellalta B. (2022). Multi-Link Operation in IEEE 802.11be WLANs. IEEE Wirel. Commun..

[5] Murti W., Yun J.-H. (2022). Multilink Operation in IEEE 802.11be Wireless LANs: Backoff Overflow Problem and Solutions. Sensors.

[6] Giordano L.G., Jonsson A. (2023). Performance and Coexistence Evaluation of IEEE 802.11be Multi-link Operation. 2023 IEEE Wireless Communications and Networking Conference (WCNC).

[7] Abdalhafid A.A., Subramaniam S.K., Zukarnain Z., Ayob F.H. (2024). Multi-Link Operation in IEEE 802.11be Extremely High Throughput: A Survey. IEEE Access.

[8] García-Rodríguez A., López-Pérez D., Galati-Giordano L., Geraci G. (2021). IEEE 802.11be: Wi-Fi 7 Strikes Back. IEEE Commun. Mag..

[9] Liu C. (2020). Discussions on PAPR Reduction Methods for DUP Mode. IEEE 802.11-20/1206r0. https://mentor.ieee.org/802.11/dcn/20/11-20-1269-35-00be-sep-nov-tgbe-teleconference-agendas.docx.

[10] Berkeley Nucleonics Corporation (2021). Wi-Fi 6 Solutions: Crest Factor and CCDF Analysis. Application Note. Berkeley Nucleonics. https://old.berkeleynucleonics.com/sites/default/files/products/resources/wi-fi_6_solutions_-_crest_factor.pdf.

[11] Sarbu A., Bechet A., Balan T., Robu D., Bechet P., Miclaus S. (2019). Using CCDF statistics for characterizing the radiated power dynamics in the near field of a mobile phone operating in 3G+ and 4G+ communication standards. Measurement.

[12] Miclaus S., Deaconescu D.B., Vatamanu D., Buda A.M. (2023). An Exposimetric Electromagnetic Comparison of Mobile Phone Emissions: 5G versus 4G Signals Analyses by Means of Statistics and Convolutional Neural Networks Classification. Technologies.

[13] Sarbu A., Miclaus S., Digulescu A., Bechet P. (2020). Comparative analysis of user exposure to the electromagnetic radiation emitted by the 4th and 5th generations of WiFi communication devices. Int. J. Environ. Res. Public Health.

[14] Miclaus S., Deaconescu D.B., Vatamanu D., Buda A.M. Mobile Phone Emissions in 5G-FR1: Using Statistic Inferences and Deep Learning for Empiric Features Extraction. Proceedings of the IEEE International Symposium on Measurements and Networking.

[15] International Commission on Non-Ionizing Radiation Protection (ICNIRP) (2020). Guidelines for Limiting Exposure to Electromagnetic Fields (100 kHz to 300 GHz). Health Phys..

[16] (2019). IEEE Standard for Safety Levels with Respect to Human Exposure to Electric, Magnetic, and Electromagnetic Fields, 0 Hz to 300 GHz.

[17] Foster K.R., Moulder J.E. (2013). WiFi and Health: Review of Current Status of Research. Health Phys..

[18] Gallucci S., Bonato M., Benini M., Chiaramello E., Fiocchi S., Tognola G., Parazzini M. (2023). Assessment of EMF Hu-man Exposure Levels Due to Wearable Antennas at 5G Frequency Band. Sensors.

[19] Jamshed M.A., Héliot F., Brown T. (2020). A Survey on Electromagnetic Risk Assessment and Evaluation Mechanism for Future Wireless Communication Systems. IEEE J. Electromagn. RF Microw. Med. Biol..

[20] ITU-T (2020). K.91: Guidance on Measurement and Numerical Prediction of Electromagnetic Fields for Compliance with Human Exposure Limits for Base Stations.

[21] Peyman A., Khalid M., Calderon C. (2011). Assessment of Exposure to Electromagnetic Fields from Wireless Computer Networks (WiFi) in Schools. Health Phys..

[22] Yang Y., Vermeeren G., Verloock L., Guxens M., Joseph W. (2025). A Survey of IEEE 802.11ax WLAN Temporal Duty Cycle for the Assessment of RF Electromagnetic Exposure. Appl. Sci..

[23] Malandrino F., Chiaramello E., Parazzini M. (2022). Performance and EMF Exposure Trade-offs in Human-centric Cell-free Networks. 2022 20th International Symposium on Modeling and Optimization in Mobile, Ad Hoc, and Wireless Networks (WiOpt).

[24] Chountala C., Chareau J.-M., Baldini G., Bonavitacola F. (2022). Experimental Assessment of Electromagnetic Field Exposure from 802.11ax Devices. 2022 9th International Conference on Wireless Networks and Mobile Communications (WINCOM).

[25] Rohde & Schwarz (2023). WiFi 7 Signal Characterization and Measurement Techniques.

[26] Januszkiewicz Ł (2018). Analysis of Human Body Shadowing Effect on Wireless Sensor Networks Operating in the 2.4 GHz Band. Sensors.

[27] MacCartney G.R., Rappaport T.S., Rangan S. Rapid Fading Due to Human Blockage in Pedestrian Crowds at 5G Millimeter-Wave Frequencies. Proceedings of the GLOBECOM 2017—2017 IEEE Global Communications Conference.

